# Associations of *VEGF-C* Genetic Polymorphisms with Urothelial Cell Carcinoma Susceptibility Differ between Smokers and Non-Smokers in Taiwan

**DOI:** 10.1371/journal.pone.0091147

**Published:** 2014-03-07

**Authors:** Min-Che Tung, Ming-Ju Hsieh, Shian-Shiang Wang, Shun-Fa Yang, Shiou-Sheng Chen, Shih-Wei Wang, Liang-Ming Lee, Wei-Jiunn Lee, Ming-Hsien Chien

**Affiliations:** 1 Department of Surgery, Tungs' Taichung Metro Harbor Hospital, Taichung, Taiwan; 2 Graduate Institute of Clinical Medicine, Taipei Medical University, Taipei, Taiwan; 3 Cancer Research Center, Changhua Christian Hospital, Changhua, Taiwan; 4 Institute of Medicine, Chung Shan Medical University, Taichung, Taiwan; 5 Division of Urology, Department of Surgery, Taichung Veterans General Hospital, Taichung, Taiwan; 6 Department of Medical Research, Chung Shan Medical University Hospital, Taichung, Taiwan; 7 Division of Urology, Taipei City Hospital Renai Branch, Taipei, Taiwan; 8 Department of Urology, National Yang-Ming University School of Medicine, Taipei, Taiwan; 9 Department of Medicine, Mackay Medical College, Taipei, Taiwan; 10 Department of Urology, Wan Fang Hospital, Taipei Medical University, Taipei, Taiwan; 11 Wan Fang Hospital, Taipei Medical University, Taipei, Taiwan; The University of Hong Kong, China

## Abstract

**Background:**

Vascular endothelial growth factor (VEGF)-C is associated with lymphangiogenesis, pelvic regional lymph node metastasis, and an antiapoptotic phenotype in urothelial cell carcinoma (UCC). Knowledge of potential roles of *VEGF-C* genetic polymorphisms in susceptibility to UCC is lacking. This study was designed to examine associations between *VEGF-C* gene variants and UCC susceptibility and evaluate whether they are modified by smoking.

**Methodology/Principal Findings:**

Five single-nucleotide polymorphisms (SNPs) of *VEGF-C* were analyzed by a TaqMan-based real-time polymerase chain reaction (PCR) in 233 patients with UCC and 520 cancer-free controls. A multivariate logistic regression was applied to model associations between genetic polymorphisms and UCC susceptibility, and to determine if the effect was modified by smoking. We found that after adjusting for other covariates, individuals within the entire population and the 476 non-smokers carrying at least one A allele at *VEGF-C* rs1485766 respectively had 1.742- and 1.834-fold risks of developing UCC than did wild-type (CC) carriers. Among the 277 smokers, we found that *VEGF-C* rs7664413 T (CT+TT) and rs2046463 G (AG+GG) allelic carriers were more prevalent in UCC patients than in non-cancer participants. Moreover, UCC patients with the smoking habit who had at least one T allele of *VEGF-C* rs7664413 were at higher risk of developing larger tumor sizes (*p* = 0.021), compared to those patients with CC homozygotes.

**Conclusions:**

Our results suggest that the involvement of *VEGF-C* genotypes in UCC risk differs among smokers compared to non-smokers among Taiwanese. The genetic polymorphism of *VEGF-C* rs7664413 might be a predictive factor for the tumor size of UCC patients who have a smoking habit.

## Introduction

Urothelial cell carcinoma (UCC) is the second most common cancer and second leading cause of death among malignancies of the genitourinary tract system in the US [1]. Urothelium covers the epithelial lining of the urinary tract from the renal calyces to the bladder, and several types of UCC (bladder, renal pelvis, and ureter carcinomas) have histologic features similar to those of transitional cell carcinoma and are considered to have an analogous etiology [2]. In Taiwan, bladder cancer is the ninth leading malignancy among men and the sixteenth leading malignancy among women [3], and bladder cancer accounts for more than 90% of UCCs in both genders. The etiology of UCC is heterogeneous, involving ethnic, environmental, genetic, and dietary factors [4]. In particular, tobacco is the main risk factor for bladder cancer, as approximately 60% of male urinary tract cancers and one-third of female urinary tract cancers are attributable to cigarette smoking [5], while occupational exposure, particularly to aromatic amines, may play role in perhaps 10% of bladder cancers [6].

Many previous studies demonstrated that tumor-associated angiogenesis and lymphangiogenesis play crucial roles in tumor progression, and angiogenic and lymphangiogenic activities are frequently correlated with tumor growth, regional lymph-node metastasis, distant metastasis, and the prognosis of patients with malignant neoplasms [7], [8]. It is well known that vascular endothelial growth factor (VEGF)-A is essential in vasculogenesis and angiogenesis. VEGF-C, a member of the VEGF family, induces lymphatic endothelial proliferation and vessel enlargement and facilitates nodal metastasis [8]. Upregulation of VEGF-C was observed in many different epithelial tumors including bladder UCC [9]–[11]. Overexpression of VEGF-C in bladder UCC was shown to be significantly related to tumor size, pathological T stage, lymphatic-venous involvement, and pelvic lymph node metastasis [10]. Moreover, upregulation of VEGF-C was also shown to play an important role in cigarette smoking-associated cervical tumorigenesis [12], but the association between VEGF-C and cigarette smoking in UCC is still unknown.

Single nucleotide polymorphisms (SNPs) are a variation in the DNA sequence that occur when a nucleotide (A, T, C, or G) is changed in at least 1% of a certain population [13]. Impacts of VEGF-A polymorphisms on human bladder cancer susceptibility were identified [14], [15], but the roles of *VEGF-C* gene SNPs and cigarette smoking in UCC susceptibility and clinical features remain unknown. In this research, a case-control study was performed on five SNPs, which are located in the intron or downstream of the *VEGF-C* gene. Some of these SNPs were reported to be correlated with the risk of oral [16] and liver cancers [17]. In this study, we examined the association between polymorphisms within the *VEGF-C* gene and UCC risk as well as the potential of an effect modification by cigarette smoking in a case-control study in Taiwanese patients.

## Materials and Methods

### Subjects and Specimen Collection

In 2011∼2012, we recruited 233 patients (with a mean age of 68.6±11.8 years) at Taichung Veterans General Hospital (Taichung, Taiwan) as a case group. All patients had pathologically proven UCC of the upper urinary tract or bladder. For the control group, we randomly chose 520 non-cancer individuals (with a mean age of 52.4±14.7 years) who were from the same geographic area. Personal information and characteristics were collected from study subjects using interviewer-administered questionnaires that contained questions involving demographic characteristics and the status of cigarette smoking. Medical data of the cases, including TNM clinical staging, the primary tumor size, lymph node involvement, and histologic grade, were obtained from their medical records. UCC patients were clinically staged at the time of diagnosis according to the TNM staging system of the American Joint Committee on Cancer (AJCC) Staging Manual (7th ed.). Tumors were classified as superficial tumors (pT0∼1, *n* = 142) or invasive tumors (pT2∼4, *n* = 91). Metastasis into lymph nodes was detected in 21 cases (9.0%), and four patients (1.7%) had distal metastasis. Before conducting this study, approval from the Institutional Review Board of Taichung Veterans General Hospital was obtained (IRB no. CF11094), and informed written consent was obtained from each individual. The whole-blood specimens collected from controls and UCC patients were placed in tubes containing ethylenediaminetetraacetic acid (EDTA), immediately centrifuged, and then stored at −80°C.

### Selection of *VEGF-C* Polymorphisms

**Figure 1:**
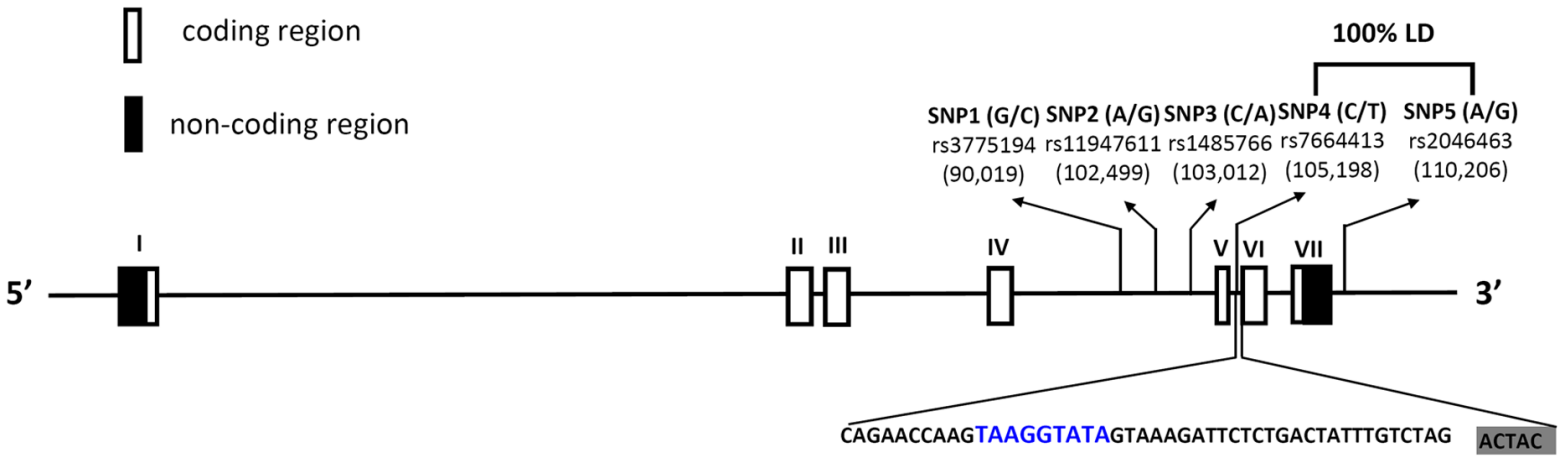
Vascular endothelial growth factor (*VEGF*)*-C* gene, locations of the genotyped variants. Schematic presentation of the *VEGF-C* (gene ID: 7424); indicating locations of the analyzed variants (rs3775194, rs11947611, rs1485766, rs7664413, and rs2046463). The blue color reveals the PESS sequence in intron 5 predicted by the PESXs server. The shading indicates the exon sequence. LD, linkage disequilibrium.

In the dbSNP database, more than 60 SNPs have been documented in the intron or downstream of the *VEGF-C* gene region. To obtain adequate power for evaluating the potential association, we investigated rs3775194, rs11947611, rs1485766, rs7664413, and rs2046463, as they have minor allelic frequencies of ≥5%. The locations of these variants were shown in Figure 1. Furthermore, these SNPs of the *VEGF-C* gene were selected in this study since they were found in cancer patients [16]–[18].

### Genomic DNA Extraction

Genomic DNA was extracted using QIAamp DNA blood mini kits (Qiagen, Valencia, CA, USA) following the manufacturer’s instructions. We dissolved DNA in TE buffer (10 mM Tris at pH 7.8 and 1 mM EDTA) and then quantified it by measuring the optical density at 260 nm. The final preparation was stored at −20°C and used to create templates for the polymerase chain reaction (PCR).

### Real-time PCR

Allelic discrimination of rs3775194, rs11947611, rs1485766, rs7664413, and rs2046463 polymorphisms of the *VEGF-C* gene was assessed with the ABI StepOne™ Real-Time PCR System (Applied Biosystems, Foster City, CA, USA) and analyzed using SDS v3.0 software (Applied Biosystems), with a TaqMan assay. The FAM respective primers used for analysis of rs3775194, rs11947611, rs1485766, rs7664413, and rs2046463 gene polymorphisms were FAM-5′-ATTTAGCACTATTAACTTCAAG, FAM-5′-TTACTTTTGAGAATGTCA, FAM-5′-CTTTTTGATTGCAGTGTTA, FAM-5′-CTTTACTATACTTTACTTGG, and FAM-5′-TTTAGCACACGGTTTAGT. The final volume for each reaction was 5 µL, containing 2.5 µL TaqMan Genotyping Master Mix, 0.125 µL TaqMan probe mix, and 10 ng genomic DNA. The real-time PCR included an initial denaturation step at 95°C for 10 min, followed by 40 cycles of 95°C for 15 s and 60°C for 1 min.

### Statistical Analyses

Differences between groups were considered significant for *p* values of <0.05. Hardy-Weinberg equilibrium (HWE) was assessed using a goodness-of-fit Χ^2^-test for biallelic markers. The Mann-Whitney *U*-test and Fisher’s exact test were used to compare differences in demographic characteristic distributions between the healthy control group and UCC patients. Adjusted odds ratios (AORs) and 95% confidence intervals (CIs) of the association of genotype frequencies with risk and clinicopathological characteristics were estimated using multiple logistic regression models after controlling for other covariates. We analyzed all data with Statistical Analytic System (SAS Institute, Cary, NC, USA) software (vers. 9.1, 2005) for Windows.

## Results

The statistical analysis of demographic characteristics is shown in Table 1. There were significantly different distributions of gender (*p<*0.001) and age (*p<*0.001) between the healthy controls and UCC patients. Hence, the AORs and 95% CIs were estimated by multiple logistic regression models after controlling for these confounders in each comparison.

**Table 1:** Distributions of demographic characteristics in 520 controls and 233 patients with urothelial cell carcinoma (UCC).

Variable	Controls (*N* = 520)	Patients (*N* = 233)	*p* value
**Age (years)**	**Mean ± S.D.**	**Mean ± S.D.**	
	52.43±14.67	68.55±11.82	*p*<0.001
**Gender**	***n*** ** (%)**	***n*** ** (%)**	
Male	426 (81.9%)	149 (63.9%)	
Female	94 (18.1%)	84 (36.1%)	*p*<0.001
**Tobacco consumption (Male)**			
No	224 (52.6%)	84 (56.4%)	
Yes	202 (47.4%)	65 (43.6%)	*p* = 0.424
**Tobacco consumption (Female)**			
No	86 (91.5%)	82 (97.6%)	
Yes	8 (8.5%)	2 (2.4%)	*p* = 0.076
**Stage**			
Superficial tumor (pTa∼pT1)		142 (60.9%)	
Invasive tumor (pT2∼pT4)		91 (39.1%)	
**Tumor T status**			
T0		65 (27.9%)	
T1∼T4		168 (72.1%)	
**Lymph node status**			
N0		212 (91.0%)	
N1+N2		21 (9.0%)	
**Metastasis**			
M0		229 (98.3%)	
M1		4 (1.7%)	
**Histopathologic grading**			
Low grade		36 (15.5%)	
High grade		197 (84.5%)	

Mann-Whitney U-test or Fisher’s exact test was used between healthy controls and patients with UCC.

In our recruited control group, frequencies of *VEGF-C* rs3775194 (*p* = 0.696, χ^2^ value: 0.153), rs11947611 (*p* = 0.092, χ^2^ value: 2.833), rs1485766 (*p* = 0.292, χ^2^ value: 1.110), rs7664413 (*p* = 0.403, χ^2^ value: 0.699), and rs2046463 (*p* = 0.403, χ^2^ value: 0.699) were in HWE. The reconstructed linkage disequilibrium (LD) plot of the five SNPs has been shown in our previous study. We found one observed haploblock in which rs7664413 and rs2046463 showed 100% LD in that study [16]. Genotype distributions and associations between UCC and *VEGF-C* gene polymorphisms are shown in Table 2. Alleles with the highest distribution frequency for the rs3775194, rs11947611, rs1485766, rs7664413, and rs2046463 genes of *VEGF-C* for both our recruited UCC patients and healthy control were respectively homozygous for G/G, heterozygous for A/G, heterozygous for C/A, homozygous for C/C, and homozygous for A/A. After adjusting for several variables, there was no significant difference in having UCC in individuals with rs3775194, rs11947611, rs7664413, and rs2046463 polymorphisms of the *VEGF-C* gene compared to wild-type (WT) individuals. However, subjects with the *VEGF-C* polymorphic rs1485766 CA, AA, and the combination of CA and AA genotypes respectively exhibited significantly (*p*<0.05) higher risks of 1.494- (95% CI: 1.051∼2.345), 2.494- (95% CI: 1.423∼4.373), and 1.742-fold (95% CI: 1.143∼2.654) of having UCC compared to their corresponding WT homozygotes.

**Table 2:** Distribution frequency of vascular endothelial growth factor (VEGF)-C genotypes in 520 controls and 233 patients with urothelial cell carcinoma (UCC).

Variable	Controls (*N* = 520) *n* (%)	Patients (*N* = 233) *n* (%)	OR (95% CI)	AOR (95% CI)
**rs3775194**				
GG	372 (71.5%)	175 (75.1%)	1.00	1.00
GC	137 (26.4%)	56 (24.0%)	0.869 (0.607∼1.244)	0.862 (0.546∼1.360)
CC	11 (2.1%)	2 (0.9%)	0.386 (0.085∼1.762)	0.329 (0.060∼1.801)
GC+CC	148 (28.5%)	58 (24.9%)	0.833 (0.585∼1.185)	0.811 (0.519∼1.265)
**rs11947611**				
AA	221 (42.5%)	93 (39.9%)	1.00	1.00
AG	249 (47.9%)	114 (48.9%)	1.088 (0.783∼1.511)	0.841 (0.559∼1.265)
GG	50 (9.6%)	26 (11.2%)	1.236 (0.726∼2.104)	1.538 (0.780∼3.031)
AG+GG	299 (57.5%)	140 (60.1%)	1.113 (0.812∼1.524)	0.929 (0.628∼1.374)
**rs1485766**				
CC	190 (36.5%)	63 (27.0%)	1.00	1.00
CA	239 (46.0%)	115 (49.4%)	**1.451 (1.011∼2.083)** *	**1.494 (1.051∼2.345)** *
AA	91 (17.5%)	55 (23.6%)	**1.823 (1.174∼2.829)** *	**2.494 (1.423∼4.373)** *
CA+AA	330 (63.5%)	170 (73.0%)	**1.554 (1.106∼2.182)** *	**1.742 (1.143∼2.654)** *
**rs7664413**				
CC	309 (59.4%)	132 (56.7%)	1.00	1.00
CT	188 (36.2%)	89 (38.2%)	1.108 (0.801∼1.533)	1.282 (0.847–1.939)
TT	23 (4.4%)	12 (5.1%)	1.221 (0.590∼2.527)	1.146 (0.423–3.103)
CT+TT	211 (40.6%)	101 (43.3%)	1.121 (0.820∼1.532)	1.267 (0.849–1.889)
**rs2046463**				
AA	309 (59.4%)	132 (56.7%)	1.00	1.00
AG	188 (36.2%)	89 (38.2%)	1.108 (0.801∼1.533)	1.282 (0.847∼1.939)
GG	23 (4.4%)	12 (5.1%)	1.221 (0.590∼2.527)	1.146 (0.423∼3.103)
AG+GG	211 (40.6%)	101 (43.3%)	1.121 (0.820∼1.532)	1.267 (0.849∼1.889)

Odds ratios (ORs) and their 95% confidence intervals (CIs) were estimated by logistic regression models. Adjusted ORs (AORs) and their 95% CIs were estimated by multiple logistic regression models after controlling for age, gender, and tobacco consumption.

*p<0.05, statistically significant.

Regardless of whether one was male or female, cigarette smoking is the main risk factor for urinary tract cancer [5], and cigarette smoking was reported to stimulate VEGF-C expression in other epithelial tumors [12]. In this study, we divided our recruited cohort into smoking and non-smoking groups and further investigated the difference between *VEGF-C* SNPs and UCC susceptibility in these two groups. In the non-smoking group, subjects with the *VEGF-C* polymorphic rs1485766 CA, AA, and the combination of CA and AA genotypes exhibited significantly (*p*<0.05) higher risks of 1.983- (95% CI: 1.320∼2.802), 2.486- (95% CI: 1.251∼4.938), and 1.834-fold (95% CI: 1.085∼3.100), respectively, of having UCC compared to their corresponding WT homozygotes (Table 3). The distribution frequency of *VEGF-C* genotypes in the non-smoking cohort was similar to the overall cohort we recruited. However, in the smoking cohort, there was no significant difference in having UCC in individuals with rs1485766 polymorphisms of the *VEGF-C* gene compared to wild-type (WT) individuals (Table 4). Among 277 smokers, subjects with the *VEGF-C* polymorphic rs7664413 CT and the combination of CT and TT genotypes respectively exhibited significantly (*p*<0.05) higher risks of 3.730- (95% CI: 1.384∼10.056) and 3.595-fold (95% CI: 1.359∼9.512) of having UCC compared to their corresponding WT homozygotes. Moreover, a similar result was also observed in subjects with the *VEGF-C* polymorphic rs2046463 (Table 4).

**Table 3:** Distribution frequency of vascular endothelial growth factor (VEGF)-C genotypes in 310 controls and 166 patients with urothelial cell carcinoma (UCC) without tobacco consumption.

Variable	Controls (*N* = 310) *n* (%)	Patients (*N* = 166) *n* (%)	OR (95% CI)	AOR (95% CI)
	Among non-tobacco consumers (*n* = 476)			
**rs3775194**				
GG	232 (74.8%)	126 (75.9%)	1.00	1.00
GC	69 (22.3%)	38 (22.9%)	1.014 (0.646∼1.593)	0.859 (0.488∼1.514)
CC	9 (2.9%)	2 (1.2%)	0.409 (0.087∼1.923)	0.411 (0.071∼2.390)
GC+CC	78 (25.2%)	40 (24.1%)	0.944 (0.609∼1.464)	0.810 (0.467∼1.406)
**rs11947611**				
AA	128 (41.3%)	68 (41.0%)	1.00	1.00
AG	152 (49.0%)	82 (49.4%)	1.015 (0.682∼1.512)	0.729 (0.438∼1.211)
GG	30 (9.7%)	16 (9.6%)	1.004 (0.512∼1.970)	1.469 (0.625∼3.453)
AG+GG	182 (58.7%)	98 (59.0%)	1.014 (0.691∼1.487)	0.824 (0.509∼1.335)
**rs1485766**				
CC	124 (40.0%)	42 (25.3%)	1.00	1.00
CA	134 (43.2%)	87 (52.4%)	**1.917 (1.232∼2.982)** *	**1.983 (1.320∼2.802)** *
AA	52 (16.8%)	37 (22.3%)	**2.101 (1.215∼3.633)** *	**2.486 (1.251∼4.938)** *
CA+AA	186 (60.0%)	124 (74.7%)	**1.968 (1.297∼2.987)** *	**1.834 (1.085∼3.100)** *
**rs7664413**				
CC	177 (57.1%)	100 (60.2%)	1.00	1.00
CT	120 (38.7%)	58 (34.9%)	0.856 (0.575∼1.274)	1.122 (0.677∼1.862)
TT	13 (4.2%)	8 (4.8%)	1.089 (0.437∼2.717)	1.188 (0.335∼4.211)
CT+TT	133 (42.9%)	66 (39.8%)	0.878 (0.598∼1.289)	1.129 (0.692∼1.841)
**rs2046463**				
AA	177 (57.1%)	100 (60.2%)	1.00	1.00
AG	120 (38.7%)	58 (34.9%)	0.856 (0.575∼1.274)	1.122 (0.677∼1.862)
GG	13 (4.2%)	8 (4.8%)	1.089 (0.437∼2.717)	1.188 (0.335∼4.211)
AG+GG	133 (42.9%)	66 (39.8%)	0.878 (0.598∼1.289)	1.129 (0.692∼1.841)

Odds ratios (ORs) and their 95% confidence intervals (CIs) were estimated by logistic regression models. Adjusted ORs (AORs) and their 95% CIs were estimated by multiple logistic regression models after controlling for age and gender.

*p<0.05, statistically significant.

**Table 4:** Distribution frequencies of vascular endothelial growth factor (VEGF)-C genotypes in 210 controls and 67 patients with urothelial cell carcinoma (UCC) who consumed tobacco.

Variable	Controls (*N* = 210) *n* (%)	Patients (*N* = 67) *n* (%)	OR (95% CI)	AOR (95% CI)
	Among tobacco consumers (*n* = 277)			
**rs3775194**				
GG	140 (66.7%)	49 (73.1%)	1.00	1.00
GC	68 (32.4%)	18 (26.9%)	0.756 (0.410∼1.396)	0.998 (0.391∼2.549)
CC	2 (0.9%)	0 (0%)	–	–
GC+CC	70 (33.3%)	18 (26.9%)	0.735 (0.399∼1.354)	0.966 (0.379∼2.462)
**rs11947611**				
AA	93 (44.3%)	25 (37.3%)	1.00	1.00
AG	97 (46.2%)	32 (47.8%)	1.227 (0.677∼2.226)	1.216 (0.497∼2.973)
GG	20 (9.5%)	10 (14.9%)	1.860 (0.773∼4.476)	3.819 (0.694∼21.027)
AG+GG	117 (55.7%)	42 (62.7%)	1.335 (0.759∼2.350)	1.412 (0.595∼3.353)
**rs1485766**				
CC	66 (31.4%)	21 (31.3%)	1.00	1.00
CA	105 (50.0%)	28 (41.8%)	0.838 (0.440∼1.596)	2.168 (0.771∼6.093)
AA	39 (18.6%)	18 (26.9%)	1.451 (0.690∼3.051)	3.219 (0.912∼15.587)
CA+AA	144 (68.6%)	46 (68.7%)	1.004 (0.555∼1.816)	2.671 (0.930∼6.929)
**rs7664413**				
CC	132 (62.9%)	32 (47.8%)	1.00	1.00
CT	68 (32.4%)	31 (46.3%)	**1.818 (1.059∼3.339)** *	**3.730 (1.384∼10.056)** *
TT	10 (4.7%)	4 (5.9%)	1.650 (0.486∼5.601)	2.377 (0.227∼24.862)
CT+TT	78 (37.1%)	35 (52.2%)	**1.851 (1.062∼3.225)** *	**3.595 (1.359∼9.512)** *
**rs2046463**				
AA	132 (62.9%)	32 (47.8%)	1.00	1.00
AG	68 (32.4%)	31 (46.3%)	**1.818 (1.059∼3.339)** *	**3.730 (1.384∼10.056)** *
GG	10 (4.7%)	4 (5.9%)	1.650 (0.486∼5.601)	2.377 (0.227∼24.862)
AG+GG	78 (37.1%)	35 (52.2%)	**1.851 (1.062∼.225)** *	**3.595 (1.359∼9.512)** *

Odds ratios (ORs) and their 95% confidence intervals (CIs) were estimated by logistic regression models. Adjusted ORs (AORs) and their 95% CIs were estimated by multiple logistic regression models after controlling for age and gender.

*p<0.05, statistically significant.

The impact of the *VEGF-C* polymorphic genotype on the pathological development of UCC was initially estimated by comparing frequency distributions of polymorphic genotypic subgroups and WT genotypic subgroups in UCC patients who had progressed to different clinical statuses including TNM clinical staging, primary tumor size, lymph node involvement, distant metastasis, and histologic grade. The comparison showed that the WT or polymorphic genotypes of rs3775194, rs11947611, and rs1485766 SNPs in UCC patients were irrelevant to the development of any UCC clinical pathological variable (data not shown). However, among UCC patients with the smoking habit, those who had the rs7664413 CT/TT SNP or rs2046463 AG/GG SNP had a higher risk (3.375-fold) of developing larger tumor sizes (*p* = 0.021) than did WT patients (Table 5 and Table S1).

**Table 5:** Distribution frequencies of the clinical status and vascular endothelial growth factor (VEGF)-C rs7664413 genotype frequencies in urothelial cell carcinoma (UCC) patients with or without tobacco consumption.

	Among non-tobacco consumers (*n* = 166)				Among tobacco consumers (*n* = 67)			
Variable	CC (*N* = 100) *n* (%)	CT+TT (*N* = 66) *n* (%)	OR (95% CI)	*p* value	CC (*N* = 32) *n* (%)	CT+TT (*N* = 35) *n* (%)	OR (95% CI)	*p* value
**Stage**								
Superficial tumor (pTa∼pT1)	54 (54.0%)	45 (68.2%)	1.00		21 (65.6%)	22 (62.9%)	1.00	
Invasive tumor (pT2∼pT4)	46 (46.0%)	21 (31.8%)	0.548 (0.286∼1.050)	0.068	11 (34.4%)	13 (37.1%)	1.128 (0.415∼3.070)	0.813
**Tumor T status**								
T0	21 (21.0%)	20 (30.3%)	1.00		16 (50.0%)	8 (22.9%)	1.00	
T1∼T4	79 (79.0%)	46 (69.7%)	0.611 (0.300∼1.246)	0.174	16 (50.0%)	27 (77.1%)	**3.375 (1.181∼9.645)**	0.021*
**Lymph node status**								
N0	89 (89.0%)	62 (93.9%)	1.00		28 (87.5%)	33 (94.3%)	1.00	
N1+N2	11 (11.0%)	4 (6.1%)	0.522 (0.159∼1.715)	0.277	4 (12.5%)	2 (5.7%)	0.424 (0.072∼2.492)	0.331
**Metastasis**								
M0	100 (100%)	65 (98.5%)	1.00		31 (96.9%)	33 (94.3%)	1.00	
M1	0 (0%)	1 (1.5%)	–	0.217	1 (3.1%)	2 (5.7%)	1.879 (0.162∼21.772)	0.609
**Histopathologic grading**								
Low grade	10 (10.0%)	13 (19.7%)	1.00		7 (21.9%)	6 (17.1%)	1.00	
High grade	90 (90.0%)	53 (80.3%)	0.453 (0.186∼1.105)	0.077	25 (78.1%)	29 (82.9%)	1.353 (0.402∼4.559)	0.625

## Discussion

Overexpression of VEGF-C was found in many epithelial tumors, including bladder UCC [9]–[11]. Recently, some *VEGF-C* SNPs (rs7664413 and rs1485766) were reported to be correlated with the risk of oral and liver cancer [16], [17], osteonecrosis of the femoral head [19], and the survival rate from ovarian cancer [18]. However, to our knowledge, there are no reports concerning the roles of *VEGF-C* SNPs in UCC. In this study, we examined the association of UCC risk with variations in *VEGF-C* and its interaction with subjects who smoke in a Taiwanese population.

The data demonstrated that subjects with the *VEGF-C* rs1485766 polymorphism were at a significantly elevated risk for UCC, suggesting that this polymorphism may contribute to the etiology of UCC. A similar finding was observed in our previous report which indicated an association between a *VEGF-C* rs1485766 polymorphism and the risk of liver cancer [17]. Since cigarette smoking is an important risk factor for UCC, we further explored any possible interactions of *VEGF-C* SNPs with smoking. We divided our recruited cohort into smoking and non-smoking groups and found that the distribution frequencies of VEGFC genotypes were similar between non-smoking groups and the overall cohort. Individuals from non-smoking groups or the overall cohort carrying the *VEGF-C* polymorphic rs1485766 CA, AA, or the combination of CA and AA genotypes had a higher risk of UCC compared to the WT genotype. The rs1485766 SNP is located in the intron 4 region of the *VEGF-C* gene (Figure 1), but the functional importance of this SNP to VEGF-C expression is still unknown. A previous report indicated that if the intronic SNP of a gene was detected in the RNA extract, the allelic gene expression with intronic SNPs gives very similar estimates to those obtained with exonic SNPs [20]. Several reports also indicated that an intronic SNP can affect gene expressions [21], [22]. We assumed that *VEGF-C* messenger (m)RNA levels might also be affected by this intronic SNP. Higher mRNA levels might translate into higher protein levels; but, as yet, we have no proof of this assumption.

Cigarette smoking induction of VEGF-A and VEGF-C expressions was demonstrated [12], [23]. Recent molecular biological studies demonstrated that the risk of UCC bladder cancer due to cigarette smoking is precisely linked to genetic markers that were detected using a microarray analysis or the SNP method [24], [25]. Different from the non-smoking cohort, we observed no significant associations between *VEGF-C* rs1485766 polymorphisms and UCC risk in the smoking cohort in our study. In smokers, a significant association was found between two polymorphisms (rs7664413 and rs2046463) of the *VEGF-C* gene and increased UCC risks. In addition to predictions of UCC risks, we also found that individuals from the smoker cohort who carried the *VEGF-C* rs7664413 CT/TT or rs2046463 AG/GG (data not shown) SNP had a higher risk of an advanced T status. Indeed, the expression level of VEGF-C was reported to be correlated with the tumor size in UCC [10], and downregulation of VEGF-C was demonstrated to decelerate tumor growth in vivo [26]. These results suggest the existence of a gene-smoking interaction in modulating the risk and tumor growth of UCC, and the effects of the *VEGF-C* rs7664413 and rs2046463 polymorphisms were highly dependent on smoking exposure. However, the mechanisms underlying the observed gene-smoking interaction remain to be elucidated.

Among different VEGFs, VEGF-A has the most complicated alternative RNA splicing (AS). As a result of AS, at least four transcripts encoding mature monomeric VEGF containing 121, 165, 189, and 206 amino acid residues (VEGF121, VEGF165, VEGF189, and VEGF206) have been detected. Each of the resulting isoforms has unique properties with pro- or antiangiogenic functions [27]. Relative to VEGF-A, other VEGFs, such as VEGF-B and -D have very few splicing isoforms, and the existence of VEGF-C splicing isoforms were only recently defined [28]. The *VEGF-C* rs7664413 SNP is located on the intron 5 flanking region (−33 nt upstream) of the *VEGF-C* gene (Figure 1). Many AS cis-regulated elements are located in this region [29]. We further found that the rs7664413 SNP was located in a sequence of a putative exonic splicing silencer (PESS; TAAGGTATA) (Figure 1). PESSs are cis-regulatory elements that inhibit the use of adjacent splice sites by acting through interactions with members of the heterogeneous nuclear ribonucleoprotein (hnRNP) family and often contribute to AS. PESSs regulate AS by recruiting factors that directly interfere with the splicing machinery [30]. For example, hnRNP I/PTB binds many exonic splicing silencers and appears to block access to the splicing machinery through protein multimerization [31]. Other evidence supports this observation about two splicing variants (ENST00000280193 and ENST00000507638) reported in the Ensemble database (vers. GRCh37). One encodes the functional VEGF-C protein (NM_005429, 420 amino acids), while the other only processes transcripts. Those data suggest that the rs7664413 SNP might affect *VEGF-C* mRNA splicing. However, further specifically designed studies are needed to verify the effects and underlying mechanism of cigarette smoking and polymorphic rs7664413 on pre-mRNA splicing. The rs2046463 SNP is located downstream of the *VEGF-C* gene but close to rs7664413 (Figure 1). We have already determined one LD haploblock constituted of rs7664413 and rs2046463 [16], which likely represent dependent genetic signals that affect the risk and tumor growth for UCC patients with a smoking habit, while other SNPs are outside the haploblock.

Our study is not without limitations. The principal limitation of this study is the relatively small sample size for gene-environment interaction assessment. Although the study power was sufficient to determine the association of the entire group of UCC patients; however, the subgroups that were analyzed were small. Therefore, conclusions drawn from these subanalyses must be interpreted with caution. Further limitation of the study might be the fact that we adjusted ORs just for age and gender without taking in consideration other potential confounding factors. In our future study, increasing the specimen number and taking more UCC risk factors into account in the analysis might precisely validate these findings. Finally, because this study is restricted to the Taiwanese population (Asian or Chinese ethnicity), it is uncertain whether these results can be generalized to other populations, and additional studies of these polymorphisms in different ethnic populations are necessary to confirm our results.

In summary, this is the first study, to our knowledge, of associations among *VEGF-C* gene polymorphisms, cigarette smoking, and UCC risk. Our findings suggest that the *VEGF-C* polymorphic rs1485766 CA/AA genotype might increase the risk for UCC, even in patients without a smoking habit. However, in association with smoking, both the rs7664413 CT/TT and rs2046463 AG/GG genotypes significantly increased individual susceptibility to UCC. After adjusting for other confounding factors, UCC patients with the *VEGF-C* polymorphic rs7664413 CT/TT genotype or rs2046463 AG/GG genotype had a higher risk of developing larger tumor sizes compared to those with the C/C or A/A homozygote.

## Supporting Information

Table S1 **Distribution frequencies of the clinical status and vascular endothelial growth factor (VEGF)-C rs2046463 genotype frequencies in urothelial cell carcinoma (UCC) patients with or without tobacco consumption.**
(DOCX)
