# Supplementary material for: Associations of VEGF-C Genetic Polymorphisms with Urothelial Cell Carcinoma Susceptibility Differ between Smokers and Non-Smokers in Taiwan
Source: PLoS One. 2014 Mar 7;9(3):e91147. doi: 10.1371/journal.pone.0091147 (PMC3946732; doi:10.1371/journal.pone.0091147)
Supplement: Table S1 — Distribution frequencies of the clinical status and vascular endothelial growth factor (VEGF)-C rs2046463 genotype frequencies in urothelial cell carcinoma (UCC) patients with or without tobacco consumption. (DOCX) [file pone.0091147.s001.docx]

**Table S1.** Distribution frequencies of the clinical status and vascular endothelial growth factor (VEGF)-C rs2046463 genotype frequencies in urothelial cell carcinoma (UCC) patients with or without tobacco consumption

|  | **Among non-tobacco consumers (*n*=166) Among tobacco consumers (*n*=67)** | | | | | | | |
| --- | --- | --- | --- | --- | --- | --- | --- | --- |
| Variable | AA (*N*=100)  *n* (%) | AG+GG (*N*=66) *n* (%) | OR (95% CI) | *p* value | AA (*N*=32)  *n* (%) | AG+GG (*N*=35) *n* (%) | OR (95% CI) | *p* value |
| **Stage** |  |  |  |  |  |  |  |  |
| Superficial tumor (pTa~pT1) | 54 (54.0%) | 45 (68.2%) | 1.00 |  | 21 (65.6%) | 22 (62.9%) | 1.00 |  |
| Invasive tumor (pT2~pT4) | 46 (46.0%) | 21 (31.8%) | 0.548 (0.286~1.050) | 0.068 | 11 (34.4%) | 13 (37.1%) | 1.128 (0.415~3.070) | 0.813 |
| **Tumor T status** |  |  |  |  |  |  |  |  |
| T0 | 21 (21.0%) | 20 (30.3%) | 1.00 |  | 16 (50.0%) | 8 (22.9%) | 1.00 |  |
| T1~T4 | 79 (79.0%) | 46 (69.7%) | 0.611 (0.300~1.246) | 0.174 | 16 (50.0%) | 27 (77.1%) | **3.375 (1.181~9.645)** | **0.021*** |
| **Lymph node status** |  |  |  |  |  |  |  |  |
| N0 | 89 (89.0%) | 62 (93.9%) | 1.00 |  | 28 (87.5%) | 33 (94.3%) | 1.00 |  |
| N1+N2 | 11 (11.0%) | 4 (6.1%) | 0.522 (0.159~1.715) | 0.277 | 4 (12.5%) | 2 (5.7%) | 0.424 (0.072~2.492) | 0.331 |
| **Metastasis** |  |  |  |  |  |  |  |  |
| M0 | 100 (100%) | 65 (98.5%) | 1.00 |  | 31 (96.9%) | 33 (94.3%) | 1.00 |  |
| M1 | 0 (0%) | 1 (1.5%) | --- | 0.217 | 1 (3.1%) | 2 (5.7%) | 1.879 (0.162~21.772) | 0.609 |
| **Histopathologic grading** |  |  |  |  |  |  |  |  |
| Low grade | 10 (10.0%) | 13 (19.7%) | 1.00 |  | 7 (21.9%) | 6 (17.1%) | 1.00 |  |
| High grade | 90 (90.0%) | 53 (80.3%) | 0.453 (0.186~1.105) | 0.077 | 25 (78.1%) | 29 (82.9%) | 1.353 (0.402~4.559) | 0.625 |
